# Examining the Impact of Corporate Social Advocacy on Customer Citizenship Behavior Among University Students: The Mediating Roles of Brand Attachment and Electronic Word-of-Mouth

**DOI:** 10.3390/bs16071093

**Published:** 2026-07-02

**Authors:** Linart Janbout, Dilber Çaglar, Pelin Bayram

**Affiliations:** 1 Faculty of Business and Economics, Girne American University, Kyrenia 99320, Türkiye; dcaglar@gau.edu.tr; 2 Faculty of Economics and Administrative Sciences, European University of Lefke, Northern Cyprus TR-10 Mersin, Lefke 99800, Türkiye; pbayram@eul.edu.tr

**Keywords:** corporate social advocacy, consumer citizenship behavior, brand attachment, electronic word of mouth

## Abstract

In this study, we examine the impact of corporate social advocacy campaigns on customer behavior, a subject which has garnered increasing interest yet remains in need of empirical evidence. We employ the Elaboration Likelihood Model (ELM) to assess the relationship between corporate social advocacy and customer citizenship behavior while considering the mediating effects of brand attachment and electronic word of mouth. Adopting a quantitative approach using Partial Least Squares–Structural Equation Modeling and a convenience sampling method, we questioned a total of 219 university-level students via a survey. University students are theoretically relevant, as they are digitally active, socially conscious, and engaged in relational rather than transactional institutional relationships, which heightens their sensitivity to advocacy initiatives and their likelihood of exhibiting extra-role behaviors. Our results show that the social advocacy of universities can directly impact the extra-role behavior of their students, which can manifest in their behaviors (i.e., helping others, providing feedback, and advocacy for the organization). These findings are beneficial for scholars as well as practitioners in academia (i.e., managers and marketing departments). Genuine, value-driven, and socially just advocacies can be enhanced with proper messaging, providing better strategic positioning for universities as socially responsible and conscious organizations, in keeping with the values of younger generations. This indicates that corporate involvement could primarily drive long-term brand success and consumer trust.

## 1. Introduction

Corporate social advocacy (hereafter CSA) is regarded as a core element of corporate strategy, with companies increasingly expected to take a stand on social and political matters. It is important to distinguish between corporate social responsibility (CSR) and CSA in that CSR entails responsible business operations and sustainability, while CSA entails the active promotion of controversial agendas, frequently spurring passionate public responses (M. D. Dodd & Supa, 2015). This is driven by growing consumer demands regarding the embodiment of ideas and values (Weber Shandwick, 2018) in organizational settings. Engaging with businesses is perceived as a social cause linked to commitment, earnestness, loyalty, and engagement among consumers. CSA is perilous by nature, risking the alienation of consumers with divergent opinions, polarizing customer bases, and resulting in reputational damage (Hong & Li, 2020). While some reports contend that CSA creates consumer trust and advocacy, others propose the contrary: CSA can prompt disengagement or a negative attitude, even more so if seen as insincere or opportunistic (Yim, 2024). This is a major driver for this study, which analyzes the impact of CSA on consumer behavior, particularly through the lens of brand attachment and electronic word of mouth (hereafter EWOM). This encompasses the provision of feedback, assisting other users/customers, and supporting the initiatives of the brand in a voluntary manner (Groth, 2005). As this research examines the education sector in Northern Cyprus, the customers are the students and the organization is the university, which can highly benefit from extra-role behaviors such as customer citizenship (Sharif & Sidi Lemine, 2024).

The literature shows that CSA can result in strong brand connections, especially when consumers align with the social causes that businesses advocate for (Rim et al., 2020). CSA can lead companies to take a stance on contentious social issues, which can be a risky yet rewarding initiative. While some individuals defend companies with CSR initiatives, others may boycott them due to a failure to identify with their position (Kull & Heath, 2016). As noted, there are contradictory findings in the literature: some scholars have shown CSA to be a stimulus for engagement (e.g., J. Y. Li et al., 2022), while others have shown that CSA can steer customers towards skepticism and/or avoidance (Yim, 2024). Despite this increased scholarly interest, CSA remains an underexplored domain with notable gaps in the extant literature (e.g., Gaither et al., 2018; Zhou & Dong, 2022), especially with regard to extra-role behavior among customers (i.e., citizenship behavior in the universities of small island developing states—SIDSs) (Sannegadu et al., 2018). Studying the psychological mechanisms that are associated with CSA and the behavioral outcomes of students in higher education can help optimize advocacy benefits while minimizing potential backlash. By addressing these gaps in the literature, our study offers valuable insights into the application of CSA in businesses, particularly for universities in smaller economies (i.e., Northern Cyprus).

Brand attachment (hereafter BA), a vital element that can influence the manner in which consumers respond to CSR practices, is the affective and psychological attachment of a consumer to a brand, influencing the consumer’s enduring commitment and loyalty (Thomson et al., 2005). A consumer who is attached to a brand will defend its advocacy efforts, including controversial ones (Fournier & Yao, 1997; Lawer & Knox, 2006). BA can have an amplifying effect on word of mouth, electronic word of mouth (EWOM), and loyalty traits when it is aligned with the consumer’s personal values (Esch et al., 2006). If, however, a brand’s CSR position is against that of the consumer, affective attachment is disrupted, and the consumer detaches from the brand or retaliates (Hong & Li, 2020; Sharif & Sidi Lemine, 2024). While prominence is given to the role of BA in consumer–brand relationships, inadequate attention is given to its moderating influence on the association between consumer satisfaction, brand CSA, and customer citizenship behaviors (CCB). Attached customers have a greater propensity for supportive behavior. Hence, our research focuses on how brands may intensify their consumers’ attachment through their advocacy, such that CSA practices do not inadvertently weaken consumer loyalty and brand reputation.

Electronic word of mouth (hereafter EWOM) is one of the crucial drivers of customers’ responses towards CSA (Sweeney et al., 2020). EWOM is defined as the phenomenon where customers express their thoughts, suggestions, and experiences of brands online (Litvin et al., 2008). These individuals express their acceptance or rejection on social media (Corcoran et al., 2016), shaping the opinions of others towards the brand. If CSA practices match consumers’ opinions, such practices have a greater chance to initiate positive EWOM through electronic means, with customers spreading positive opinions and contributing to advocacy practices. However, insincere or opposing values can lead to the spread of negative EWOM, which can negatively impact the reputation and authenticity of a brand (i.e., universities in the current case) (S. Kim & Ferguson, 2022). Despite the growing body of studies on this subject, the theoretical mechanisms by which CSA shapes consumer behavior have not been thoroughly studied. Research has mostly focused on proximal outcomes such as corporate reputation, attitudes, and purchases intentions, with limited attention to the underlying psychological processes of CSA and voluntary behaviors. Communication through CSA can improve emotional bonds with the brand, which can increase consumers’ tendency to advocate for the brand by spreading positive EWOM. This represents a significant theoretical gap in understanding CSA and relational outcomes, especially in this context.

**Figure 1:**
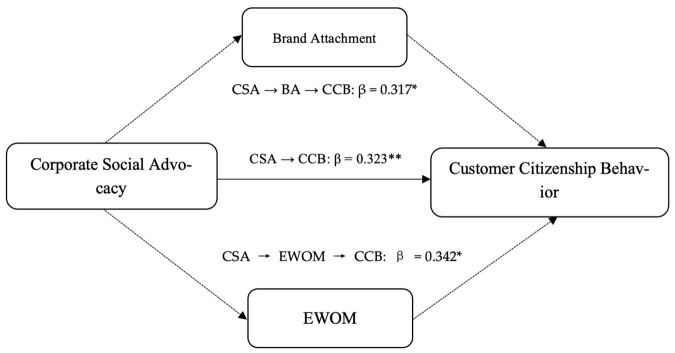
Research model. * *p* < 0.05 and ** *p* < 0.01.

CSA has been widely examined in the corporate context, especially in Western economies, which has left a gap in the understanding of such initiatives and their influence on consumer behavior in smaller regions. As universities behave as brands that engage in corporate advocacy for social, environmental, and other issues (particularly online), socially aware and tech savvy university students can be directed towards showing citizenship behaviors through sharing, recommendations, and advocacy for their universities (Gohar et al., 2023). Northern Cyprus, as an SIDS, represents a unique socio-economic environment where university students represent a digitally savvy and socially responsible demographic who can provide insights into the effects of CSA on BA and EWOM, as well as CCB (Parcha, 2024; Sannegadu et al., 2018). This demographic represents future consumers who are actively engaged with CSA, making them relevant to the current study. The underrepresentation of the contextual setting of Northern Cyprus in the literature provides the motivation for this study. University students fit this context, as their familiarity with digital platforms and sensitivity towards environmental issues make them more likely to engage with or reject the CSA efforts of brands (i.e., of universities). Beyond these demographic and behavioral characteristics, students represent an appropriate population for CSA-CCB research, as their linkage with a university is identity-based and relational, which is distinct from commercial consumer relationships. This implies that CSA initiatives are more likely to resonate at the personal value level and be associated with voluntary behaviors such as CCB. This aligns the theoretical setting with the study population and the constructs examined in this research, providing a robust rationale for this study’s focus on university students. These aspects represent geographic, demographic, and industrial gaps noted in the literature (Aljuhmani, 2019; Gohar et al., 2023), which open a pathway for additional examinations, especially in underexplored contexts (Gong & Yi, 2021). Examining these interactions using the current model (see Figure 1) will help elucidate the impact of corporate advocacy on consumer behavior in the digital realm.

While existing studies have examined brand involvement (J. Y. Li et al., 2022), corporate reputation (M. D. Dodd & Supa, 2014), public legitimacy (Health & Waymer, 2014), behavioral intentions (Overton et al., 2020; K. Park & Jiang, 2023), word of mouth (S. Kim & Ferguson, 2022; Overton et al., 2020), and brand loyalty (K. Park & Jiang, 2023), the notion of customer citizenship behavior remains scarcely addressed, as the consequences of CCB have been studied more than its causes (Gohar et al., 2023; Hong & Li, 2020; S. H. Kim et al., 2019). Understanding these mediating dynamics can provide firms with actionable solutions for taking advantage of CSA programs without compromising positive consumer engagement. The hypotheses, methods, results, and theoretical and practical implications of this study are discussed in the following sections. The paper concludes by making recommendations for future research, highlighting the significance of gauging consumer reactions to CSA in a changing sociopolitical context. Through this study, we address gaps in CSA research, contributing to a broader discussion on corporate advocacy, consumer behavior, and brand management.

## 2. Hypotheses and Theories

### 2.1. Theoretical Framework

CSA has been incorporated into corporate planning in response to increased demands on companies to take stances on social and political affairs. In contrast to CSR, which has historically comprised good business practices and sustainability, CSA involves companies taking clear stances on salient, contested topics and, in so doing, receiving fervent support from consumers and possible backlash (M. D. Dodd & Supa, 2015). The activity aligns with increased pressures on brands, and, in general, on youth, to convey social responsibility in addition to profit (Weber Shandwick, 2018). CSA is, however, a risky and multifaceted activity where companies may alienate consumers who do not share their position (J. Y. Li et al., 2022). Despite CSA’s increased saliency, the literature on CSA’s influence on consumer behavior in the longer term is scarce. The Elaboration Likelihood Model (ELM) (Petty et al., 2015) is a theory that addresses CSA message processing and its impact on consumer attitudes and behavior. The model explains the mechanisms by which people process persuasive messages, either through a central route (deep and effortful processing of information—high involvement) or a peripheral route (surface-level attractiveness or emotional tone—low involvement). In this context, CSA and EWOM are persuasive communications: university students may engage through central or peripheral routes based on their involvement with the social issue (BA), and CCB is an outcome influenced by CSA and BA. Since the ELM has notably been employed in similar contextual studies (e.g., Feng, 2024; Leong et al., 2019), the application of ELM is justified for the hypothesis development process of the current study. ELM highlights the focal constructs of this research, as CSA functions as the persuasive message and brand attachment reflects individual-level involvement and the spread of EWOM as a secondary channel. This helps in identifying determinants that can lead to citizenship behaviors among students in this context, with the central route (careful assessment of the issue–brand link) leading to a more stable and internalized attitude expressed through CCB.

ELM provides an appropriate explanatory perspective for this research, as responses to CSA are not uniform but are instead formed through various levels of message processing (Song & Choi, 2023). Under the premise of ELM, individuals tend to respond to CSA messages based on effort and reflective assessment when the subject is relevant. This can occur through affective and symbolic signals linked to motivations or the individual’s ability to elaborate on the subject (Camilleri, 2022). In the context of university students, this process is assessed through the credibility and alignment of the issue with the values of students, while remaining consistent with the identity of the university (S. Y. Lee & Chung, 2023). If the evaluation is positive, it may lead to positive internalization and responses to the institution encouraging extra-role behaviors (Yim, 2024). BA is positioned in this research as an internal relational outcome, reflecting the emotional closeness achieved by advocacy initiatives, while EWOM represents an external expression of communicative responses to such initiatives. Thus, CCB reflects a broad voluntary behavioral outcome that emerges based on positive evaluations that are internalized and adequately translated into supportive actions (Zhang & Zhou, 2023). ELM explains how the influence of CSA can unfold via BA and EWOM mechanisms. These constructs are not one-to-one representatives of ELM elements but are rather a conceptualization of CSA as a focal persuasive stimulus that encourages assessment, with BA and EWOM treated as downstream factors for how advocacy is interpreted. Under the premise of ELM, the personal relevance and motivation that drive students to take part in university advocacy initiatives are operationalized as involvement. Therefore, BA is a relational consequence that can develop through meaningful perceptions of advocacy messages and their credibility (Camilleri, 2022), which aligns the theoretical framework of the present model with that of the ELM. Notably, ELM is applied here as an interpretive lens rather than a directly assessed mechanism. In other words, central and peripheral processing routes are not operationalized as separate constructs in the measurement model but serve as conceptual anchors for variable explanations in terms of students’ responses to CSA.

### 2.2. Corporate Social Advocacy (CSA) and Customer Citizenship Behavior (CCB)

CSA is where businesses publicly take positions on sociopolitical issues despite potential reputational, resource, and stakeholder costs (M. D. Dodd & Supa, 2014, 2015). Contrary to CSR, where businesses prioritize moral business practices and sustainability, CSA involves businesses taking stands on contested issues, thus eliciting polarized consumer responses. CSA widens businesses’ conceptions of corporate citizenship: rather than responding to society passively, businesses take an active position in movements, something which not everyone may agree with (Sharif & Sidi Lemine, 2024). While, historically, businesses have avoided addressing sensitive issues in order to maintain market acceptance by everyone, CSA goes against such deep-rooted expectations of remaining neutral (M. Dodd, 2018; Sweeney et al., 2020). As CSA becomes increasingly prevalent, scholars have striven to determine its influence on consumer–brand relationships. While some have claimed that CSA boosts brand reputation and customer loyalty, particularly for consumers who identify with the brand’s cause (Johnston & Lane, 2019; Parcha, 2024), others have, conversely, argued that CSA causes resistance and negative attitudes toward brands from consumers who disagree with the position of the business (Abitbol et al., 2018). M. D. Dodd and Supa (2015) claim that CSA splits consumers into two camps: supporters, who interact positively and strengthen purchase intentions, and boycotters, who boycott and may participate in negative WOM (Leonidou & Skarmeas, 2017; Rasool et al., 2021). While CSA adoption is on the rise, its influence on consumer behavior in the long term remains underexplored.

CCB represents the discretionary, extra-role behavior of consumers in assisting a business, a business’s customers, or fellow consumers (Bove et al., 2009). CCB is an extension of transactional relationships and includes giving constructive feedback, assisting fellow customers, and WOM (i.e., promotion of the brand) (Leong et al., 2019). It is distinct from customer loyalty, as positive consumer activity and overt assistance on behalf of the business are evident. Several studies have examined antecedents to CCB, such as customer satisfaction, service involvement, and perceived value (e.g., Dalal & Aljarah, 2021; Mitrega et al., 2022). CCB is also related to CSR, as customers tend to demonstrate extra-role behaviors if they feel that an organization is socially responsible (Aljarah, 2020; Zhu et al., 2016). It is beneficial for businesses to recognize how CSA influences CCB, as it motivates age-old loyalty in the form of today’s consumers’ voluntary advocacy and protection of a brand. As corporate advocacy rises, studies on the CSA–CCB association can provide valuable insights on how businesses may better engage with consumers in depth.

CCB comprises three dimensions, as operationalized in the current study: helping, advocacy, and feedback behaviors (Yi & Gong, 2013). Notably, these dimensions are widely adopted in the extant literature and in a service context. Helping behavior can be described as a voluntary initiative by existing customers to support other customers or the brand (e.g., by responding to questions, guiding others, or sharing experiences), which can lead to a better experience for others. In the current context, students who are attached to a university and its CSA tend to assist their peers or prospective students by sharing relevant information (Fatma et al., 2022). Advocacy behavior can be defined as the degree to which an individual promotes or recommends a brand to others in accordance with their emotional attachment and positive experiences. University students can be expressive of their attachment, which can manifest as positive words about the university and its social values (van Tonder & Petzer, 2021). This, in turn, can improve the perspective and engagement of others, particularly when their values and the university’s CSA initiatives are aligned. Lastly, feedback pertains to the willingness of individuals to provide suggestions, comments, and constructive feedback to aid a brand in enhancing its services (van Tonder & Petzer, 2021). In the context of this paper, students can take proactive initiative to communicate their ideas, as they have a sense of investment in their university due to the university’s CSA practices. Notably, these dimensions are included in the current analysis, as is illustrated in the research model shown in Figure 1.

The Elaboration Likelihood Model (ELM) (Petty & Cacioppo, 1986; Petty et al., 2015) provides a theory on how consumers process CSA messages, and how said messages affect their attitudes and behaviors. ELM states that persuasion messages take two general routes: a central route, through extensive thinking and deliberation on information, and a peripheral route, through surface-level cues such as affective appeal or brand reputation (C. Y. Li, 2013). In the case of CSA, individuals concerned with specific issues tend to process corporate advocacy messages through the central route to build lasting behavior and attitudes (Parcha & Kingsley Westerman, 2020; Parcha, 2024). Less concerned individuals process CSA messages through peripheral routes, including brand persona, social signals, or affective appeals, and not through critically analyzing advocacy initiatives (Cheng, 2022; Y. Lee & Tao, 2021). As CSA is expected to address sensitive issues, the ELM establishes a strong identifier for consumers (i.e., university students) who have a better chance of showing CCB. These consumers defend and promote the brand’s behavior and thus convey the saliency and expression of CSA messages (Parcha & Kingsley Westerman, 2020; Pelletier et al., 2020). Firms can devise better advocacy marketing strategies in accordance with the market’s values and aspirations by considering the persuasion routes through which CSA messages affect consumer involvement (S. Kim & Ferguson, 2022).

In this research, we argue that CSA directly impacts extra-role behavior among university students (i.e., CCB), as they expect the university to engage in social action and take stands on ethical issues. This perception of social responsibility and value-centric action steers students towards advocacy. Helping and feedback (Fatma et al., 2022) stem from the emotional bond fostered by the alignment of CSA with students’ values, leading to the voluntary participation of students in the success of the university. Although several studies have assessed CSA and consumer responses, there are limited explanations of how advocacy translates into CCB through relational and communicative patterns, particularly in higher education settings. While this has not been ignored altogether, the behavioral consequences of CSA have been less systematically addressed for non-commercial contexts and mediating processes. In accordance with our discussion, the following hypothesis has been developed:

**Hypothesis** **1.** *Corporate social advocacy has a positive influence on customer citizenship behavior for university students in Northern Cyprus.*

### 2.3. Mediating Role of Brand Attachment

Brand attachment (BA) is defined as an enduring attachment, commitment, and loyalty towards a brand/organization and is described by psychological and affective attachment between a brand and a consumer (Schultz et al., 1989). Organizations benefit from customers having opportunities to support a firm’s advocacy, even if such advocacy is for unpopular social causes (Japutra et al., 2014). BA is a powerful determinant of the attitude and behavior of a consumer, as emotionally attached customers defend, promote, and lobby for the brand (i.e., universities in the current case) (Malär et al., 2011). The likelihood of BA mediating the linkage between CSA and CCB is supported by what has been discussed in this section. This form of attachment is shaped by emotional bonds between the customer (i.e., students) and the brand (i.e., university) due to the socially responsible commitment initiatives of the brand (Manser Payne et al., 2020). Such bonds yield loyalty and motivations beyond mere purchases (enrollment in the university), which can translate into extra-role behaviors and a sustained engagement with the brand.

While significant, BA’s role in the relationship between CSA and CCB is still unclear. While studies have established that BA promotes the attachment and activity of consumers, its direct impact on CCB following CSA remains to be fully discovered (Sharif & Sidi Lemine, 2024). An understanding of how CSA creates BA and, in so doing, influences voluntary behavior on behalf of consumers can provide companies with clear insights into building enhanced consumer–brand bonds amidst corporate advocacy movements. BA can act as a crucial mediator for the linkage between CSA and CCG, as it entails the perception of brands’ advocacy in alignment with personal values. This leads to the development of a deeper emotional bond with the organization. This, in turn, can yield motivation and engagement in voluntary and supportive behavior that goes beyond mere transactions (Ahmad et al., 2023). In the extant literature, it has been reported that BA can be a mediating variable between customers’ experience of a brand and citizenship behaviors (e.g., Ahmad et al., 2023; Shimul & Phau, 2022). Such emotional bonds guide individuals to advocate for the brand, as they emphasize personal identification with the said brand. Thus, cultivating a strong emotional link with customers (i.e., university students in this case) can enhance the effectiveness of CSA initiatives (Feng, 2024).

According to the premise of ELM (Parcha, 2024), university students process persuasive information from their university through central or peripheral routes, which can vary based on their motivation and the degree to which they engage with the message.

In the context of CSA, when university students in Northern Cyprus perceive CSA initiatives and messages as relevant and consistent with their values, they may, under the central route of ELM, be more likely to exhibit extra-role behaviors such as CCB. However, this study does not directly measure the processing route and treats this as an interpretive inference. In such scenarios, the attitude of students towards their university remains positive in a lasting manner, which creates BA. As emotional connections are formed, BA can act as a motivating factor that better explains behavioral outcomes such as supporting the brand and its messages. Thus, through an ELM lens, cognitive and emotional engagement with advocacy messages can translate into extra-role behaviors among students, who go on to show support for their universities. In light of what was discussed in this section, the following hypothesis has emerged:

**Hypothesis** **2.** *Brand attachment can mediate the relationship between CSA and CCB in the context of university students in Northern Cyprus.*

### 2.4. Mediating Role of Electronic Word of Mouth (EWOM)

Electronic word of mouth (EWOM) is the online sharing of consumers’ brand-related experiences, opinions, and recommendations (Litvin et al., 2008). With electronic media continuing to impact consumer interactions, EWOM has become an effective way of extending CSA messages. Positive EWOM can induce brand credibility and consumer trust, while negative EWOM can damage a brand’s reputation and deter consumer engagement (S. H. Kim et al., 2019). The literature asserts that CSA initiatives influence EWOM, as consumers supporting a company’s position are prone to share their opinions online (M. D. Dodd & Supa, 2015). However, EWOM’s mediating role in the CSA–CCB relationship remains underexplored. Investigating how EWOM contributes to consumer advocacy will provide pragmatic insight on how to implement corporate advocacy initiatives online. EWOM can act as a major element linking university CSA to the behavioral outcomes of students (i.e., CCB). The discussion and sharing of content is stimulated by organizations engaging in CSA initiatives, which leads to user-generated content and increased authenticity, shaping others’ perceptions and behaviors towards the said organization (Moradi et al., 2022). In a comprehensive systematic review, it was noted that EWOM has a significant impact on the decision-making processes of consumers, as it can alter their attitudes and behavioral intentions (Ismagilova et al., 2021). It is important to note that the conceptual boundary between EWOM and the advocacy dimensions of CCB involves promotional communications. EWOM, in this research, refers to the organic online sharing of experiences and opinions (Litvin et al., 2008), whereas CCB advocacy reflects a deliberate discretionary behavior of actively recommending and defending a university (Yi & Gong, 2013). The discriminant validity (HTMT) values of these two constructs confirm that they remain empirically distinct.

In addition, EWOM can also mediate the credibility of a brand and the subsequent behavioral outcomes of consumers, as it shapes the responses of potential consumers (Moradi et al., 2022). It has also been noted that younger consumers (e.g., Gen Z) tend to be more influenced by EWOM as they spend more time on social media (Kumar et al., 2023). ELM enables the current research to explain the indirect effect of EWOM on the linkage between CSA and CCB in the context of SIDS universities, as it pertains to the mental processes of individuals regarding the social advocacy of universities. In the peripheral route, students are more likely to rely on the credibility and attractiveness of the message source, while, in central route, they are more likely to scrutinize the quality and the relevance of the contents of EWOM. Ismagilova et al. (2021) reported that both ELM routes for EWOM are highly influential in terms of behavioral outcomes among consumers, which is in line with the arguments of the current research. Similar reports have been made in the extant literature, demonstrating the dependency of EWOM and its effectiveness on the route of data processing that is adopted by individuals (e.g., Moradi et al., 2022). When the quality of EWOM content is high and the source is socially proximate, both the peripheral and central routes of ELM may theoretically be implicated in shaping CCB, though this study treats such routes of engagement as a conceptual interpretation rather than an empirically verified mechanism (Kumar et al., 2023; Leong et al., 2019).

Within this context, EWOM plays a critical role as a mediator for the relationship between CSA and CCB, as it enables students to express their values and their support in the digital domain. The CSA initiatives of a university can encourage students to engage in online communication of their support, which can foster a ripple effect, leading to the development of citizenship behaviors (S. Kim & Ferguson, 2022). Thus, EWOM can lead to positive perceptions towards the brand and trigger behavioral outcomes (i.e., CCB) in the current context. Hence, the following hypothesis has emerged:

**Hypothesis** **3.** *Electronic word of mouth (EWOM) has a mediating impact on the CSA-CCB linkage among university students in Northern Cyprus.*

## 3. Research Design

### 3.1. Methodology and Approach

In our current research, we employ a quantitative approach in which the purposive sampling method is combined with a convenience sampling method to ensure that A) participants fit the context of the study (i.e., university students who are active on social media and online platforms) and B) participants are selected based on their availability and willingness to participate. Deans, ethics committee members, and other relevant decision-makers were informed of this study, and the necessary permissions were obtained to conduct this research among the university students of Northern Cyprus. Data were gathered from three (3) different universities across the state. The total registered student population across the three participating universities in Northern Cyprus at the time of data collection was approximately 15,000. G*power software version 3.1 was used to calculate the necessary sample size to maintain a statistical power of 90%, with an effect size of 0.15 and α = 0.05. These settings were combined with the recommendations for performing structural equation modeling analysis (Hair et al., 2017). The calculated sample size was between 176 and 221. To maintain the statistical significance and internal reliability of the dataset, 230 questionnaires were distributed, and a total of 219 qualified for the final analysis, with a response rate of 95% (incomplete or withdrawn responses). The survey was administered in a paper format across three universities during the spring semester of the relevant academic year. Permissions were obtained from the dean’s office and ethics committee prior to distribution. Surveys were given to students directly in common areas by the lead researcher, who remained present in order to answer procedural questions. Participation was completely voluntary, and no incentives were offered. All participants were informed of the purpose of the research and completed a written consent form. It is important to note that the study does not claim statistical representativeness of the full student population. The sampling approach was designed to achieve adequate power for the structural model and theoretical transferability within the defined context. Purposive criteria ensured that participants were active social media users and enrolled students, and convenience sampling was used to achieve the required count within university access constraints.

For the pilot test, the same manner of distribution was used for a separate group of students at one of the three universities to ensure the readability, understandability, and appropriateness of the items, with 25 respondents who were excluded from the final analysis. No items were removed or changed after the pilot test. Due to the quantitative approach of the research, deductive hypotheses have been developed that are supported by the theoretical context of the research to provide logical insights. The design, data collection procedures, identification of variables, and analytical techniques were determined based on the available literature and appropriateness for the deductive approach (Adler, 2022; Casula et al., 2021). It is important to note that this study is limited in terms of sampling design, as the sampling techniques employed are context-specific and thus have a limited generalizability (e.g., to broader higher education environments and student populations). Given the relational and socially visible nature of university student interactions in a small island context, this research provides a meaningful empirical setting for theory extension while acknowledging its limitations. While the purposive convenience sampling technique is appropriate for establishing contextual fit, it substantially constrains the external validity of the findings beyond the examined university setting. Hence, the results should be interpreted as context-specific and theoretically informative rather than generalizable across all higher education systems or broader student populations.

### 3.2. Respondents’ Profile

Table 1 below represents the demographic characteristics of the participants. Age, gender, and education level are included in the analytical technique as control variables due to their impact on the outcomes of the research (Mustafa & Kasamani, 2021; Hamzah et al., 2021).

### 3.3. Measurements

During the development of the questionnaire, various valid and commonly used measurement scales were derived from the extant literature.

To measure corporate social advocacy (CSA), four questions were derived from the work of Hambrick and Wowak (2021) and M. D. Dodd and Supa (2014). It is important to note that CSA is operationalized as students’ overall perception of their university’s social advocacy positioning and not as a mere response to specific campaigns or initiatives. This perception-based approach is consistent with the existing CSA literature from service and higher education settings (e.g., M. D. Dodd & Supa, 2015; Parcha, 2024). The ability to attribute findings to specific advocacy acts or communications is thus limited.

To assess brand attachment (BA), three questions were developed based on the work of C. W. Park et al. (2010), followed by three questions addressing electronic word of mouth (EWOM) that were derived from the works of Hennig-Thurau et al. (2004) and Cheung and Thadani (2012). Lastly, nine questions were developed (three questions for each dimension) assessing the customer citizenship behavior (CCB) of university students: questions addressing the helping dimension were derived from the work of Yi and Gong (2013); feedback behavior questions were derived from Yi and Gong (2013); and advocacy was measured based on the work of Groth (2005) and Yi and Gong (2013). All questions utilized a 5-item Likert scale ranging from 1 = strongly disagree to 5 = strongly agree. All items were adapted from established scales in the existing literature and were slightly revised (i.e., minor word adjustments) to fit the context of universities while retaining clarity and conceptual meaning (Appendix A). The previously mentioned pilot test ensured that alignment and appropriateness were reviewed prior to the full data collection stage. Procedural remedies were considered, as single-survey cross-sectional data can yield Common Method Bias (CMB) (Podsakoff et al., 2024). Data confidentiality and anonymity were ensured for each participant, and withdrawal was made available at any stage upon request. No unnecessary data were gathered to reduce Common Method Bias (CMB) (Podsakoff et al., 2024). To examine CMB, the Unmeasured Latent Method Construct (ULMC) was used for all items; the average substantial loading was 0.61 and the average method loading was 0.07 (Kock, 2015; Sarstedt et al., 2017). While this procedure ensured that CMB was minimized, this bias cannot be entirely ruled out in cross-sectional self-reported research.

### 3.4. Research Model

In the light of the objectives of the research, and in order to address the existing gaps in the literature and the theoretical framework that led to the development of the research hypotheses, the following causal model has emerged, illustrating the core analytical scope of the study. As the research draws on ELM as a theoretical foundation, central and peripheral processing are not operationalized as separate routes. Route membership is explicitly tested by applying ELM as an interpretive framework to explain students’ varying responses to CSA based on their cognitive engagement with the content or with affective and symbolic signals. Thus, the current research does not measure central and peripheral processing directly but rather uses the premise of ELM to provide a broader explanatory framework for understanding CSA assessment that can translate into attachment, EWOM, and citizenship behavior among university students. This procedure maintains model and theoretical consistency while avoiding overextension. Figure 1 presents the proposed research model in the context of Northern Cypriot universities.

## 4. Analysis and Discussion

The proposed model presented in Figure 1 was analyzed using Smart-PLS software (version 4) with Partial Least Squares–Structural Equation Modeling (PLS-SEM), as it encompasses latent variables, is not affected by normal distribution in the data, and relies on a relatively small sample size (Hair et al., 2017). As presented in Table 2 (measurement model assessment), the loading values are within the acceptable threshold of 0.7 and 0.9. The Rho A, alpha, and composite reliability measures are similarly statistically satisfactory (Jöreskog, 1971; Diamantopoulos et al., 2012; Dijkstra & Henseler, 2015; Hair et al., 2019). In addition, average variance extracted (AVE) values remain above 0.5, which shows that convergent validity is satisfactory (Hair et al., 2017). This suggests that the current proposed model is a good fit, and its parameters are adequate for further analysis. PLS-SEM is deemed appropriate due to the assessment of its predictive relationships with latent constructs with indirect effects and higher-order outcomes. CCB is modeled with dimensionality, providing a more appropriate estimate within a structural setting. As this research focuses on theory extension, mediation testing, and model prediction in a specific context, this technique provides analytical parsimony and is commonly used in consumer behavior research for objectively measuring complex latent variable relationships with measurement quality and structural path coefficients (Hair et al., 2019).

The Heterotrait–Monotrait ratio (HTMT) is calculated in Table 3, which shows that the values remain below 0.85, implying that the convergent validity for the model is acceptable (Henseler et al., 2015; Purwanto & Sudargini, 2021).

CCB is modeled as a higher-order construct using a Type II reflective–formative specification (Hair et al., 2019): the three dimensions (helping, advocacy, feedback) are reflective at the first-order level (as reported in Table 2) and formative at the second-order level (as reported in Table 4), which is consistent with the theoretical conceptualization of CCB as a composite outcome construct (Yi & Gong, 2013). Table 4 presents the outer weights, convergent validity, and VIF values for the second-order formative stage, all of which confirm acceptable validity and the absence of multicollinearity (VIF < 3; Hair et al., 2019).

Table 5 presents the hypothesis testing results. Model fit is assessed using the Standardized Root Mean Square Residual (SRMR = 0.061), which falls within the recommended threshold of <0.08 for PLS-SEM (Henseler et al., 2014). As PLS-SEM is a variance-based technique, covariance-based fit indices such as CFI and NFI are not reported (Hair et al., 2019). Both R^2^ and Q^2^ values are satisfactory, indicating adequate predictive power and relevance (Shmueli et al., 2016, 2019). Additionally, RMSE and MAE values approach zero, further confirming model fit (Shmueli et al., 2019).

While the directionality of the hypothesized relationships is consistent with theoretical predictions, the empirical value of the current research lies in the simultaneous examination of these paths within an integrated mediational framework applied to the higher education context of Northern Cyprus. This setting has not been previously examined in the CSA literature, and the standardized path coefficients confirm the model while providing a context-specific quantification of effect magnitudes.

Our findings provide a clear understanding of how CSA influences sustainable CCB among university students in Northern Cyprus. In line with the proposed model (see Figure 1), both direct and indirect paths through which CSA contributes to positive student behaviors extending above formal expectations are supported. The direct relationship between CSA and CCB is shown in Table 5 (β = 0.323, t = 4.678), which confirms the first hypothesis. This suggests that, by engaging in advocacy initiatives, universities encourage supportive behaviors that include helping others, providing constructive and positive feedback, recommending the university, and voluntarily advocating on its behalf among students (Sharif & Sidi Lemine, 2024). In the current higher education context, operations are carried out within a relatively small and socially connected setting, where such behaviors can have a strong reputational effect (Mitrega et al., 2022; Parcha, 2024). CSA can be highly effective in triggering positive behavioral outcomes among students, which can have in-depth and lasting effects on the organization and its image and reputation among young communities, particularly due to the smaller size of the economy in this state. This finding is theoretically meaningful because, in a relational, identity-based institutional context such as that of a university, particularly in a small, socially connected economy, students do not experience CSA as a distant corporate communication but as a direct expression of shared values, which produces behavioral rather than merely attitudinal responses.

Our results also show that, through CSA initiatives that are commonly supported among students at the university level, a university can enhance its image and potentially increase its market share due to the supportive behaviors of students (e.g., feedback, helping others, and advocacy for the university). This finding provides support for the second hypothesis of the research on how BA mediates the relationship between CSA and CCB (β = 0.317, t = 2.844). The confirmation of the mediating role of BA in this context between CSA and CCB implies that such initiatives can be much more effective, as they can strengthen students’ emotional bonds with the university. This leads to a better perception of the university’s genuineness in supporting social causes, reflecting shared values with the students and leading to students feeling more emotionally connected to their institution (Ahmad et al., 2023; Feng, 2024). Attachment to the organization (BA) is boosted via its CSA, which will further encourage students to become involved with the advocacy initiatives of their university, as this advocacy addresses various social aspects of life (e.g., sustainable fashion) (Jorgensen, 2021). This, in turn, increases the likelihood of students engaging in positive citizenship behaviors. Thus, in this context, BA serves as an important mechanism that translates socially responsible advocacy into emotional bonds with stronger behavioral commitments. This is particularly notable in the Northern Cyprus context, where university–student relationships are embedded in a compact socio-institutional environment: the university’s identity relevance is heightened, making brand attachment a more potent behavioral driver than it may be in larger, more anonymous higher education markets.

Lastly, our results are in line with the third hypothesis, which concerns the mediating role of EWOM on the relationship between CSA and CCB (β = 0.342, t = 2.895), particularly in the context of university students in Northern Cyprus. This mediation effect highlights the importance of student communication and peer influence within a higher education environment. As students are highly active in the digital domain and frequently share opinions, experiences, and evaluations through online platforms, CSA initiatives become an essential factor for students who may be more inclined to communicate interpersonally on digital channels. Through this mechanism, the influence of advocacy initiatives further encourages CCB by strengthening positive social diffusion around the university and amplifying EWOM (Kumar et al., 2023; Moradi et al., 2022). These initiatives can generate a spread of EWOM among students who are active on social media, and their attachment to the business (i.e., their university) can be highly beneficial for the organization, as they are more likely to exhibit proactive and voluntary loyalty and extra-role behaviors such as CCB. These results are consistent with the existing literature (Fatma et al., 2022; Jia et al., 2022); the current research contributes to the literature on CCB, EWOM, and CSA by providing empirical evidence from a sector that is underexplored (i.e., higher education institutions—universities), as the majority of studies tend to focus on other industries, such as tourism, retail and fashion, and banking (Huang et al., 2023; Mohammad Shafiee & Tabaeeian, 2022). Beyond statistical confirmation, our findings reveal that, in digitally active student populations, EWOM functions not merely as a communication channel but as a mechanism of social identity expression. Students who share positive content related to their university’s advocacy are not only amplifying its reach but are publicly aligning themselves with its values, which reinforces their own commitment to citizenship behaviors.

Furthermore, in the current research, we employed the theoretical premise of ELM (Petty et al., 2015), where CSA messages are guided and processed based on their impact and influence on the attitudes of university students who, at this level, are aware, conscious, and up to date with local, regional, and global trends (Aljarah, 2020). Our results suggest that universities may theoretically engage central and peripheral processing tendencies among students through adequately designed CSA initiatives. However, the present study does not directly measure route activation and instead relies on ELM as a framework for interpreting differential student responses (Feng, 2024).

The current findings therefore interpret, rather than confirm, the operation of these two routes in the higher education context of Northern Cyprus. EWOM can be a significant element for improving extra-role behavior among students, as online platforms can enable students to share their ideas and suggestions with groups that can be public or specific. These findings show that both emotional and communicative mechanisms connect CSA and CCB, implying that socially oriented advocacy initiatives become more effective when they create emotional attachments and stimulate positive online communication among students. This is relevant in the higher education sector, where students and the university have a relationship that is relational and identity-based rather than purely transactional. This also fits within the lens of the Elaboration Likelihood Model (ELM), as this study shows that CSA messages may function as persuasive stimuli, influencing students through deeper cognitive processing or through social and emotional cues. When students are more involved in social issues, they respond more strongly to the content and authenticity of a university’s advocacy initiatives, while others react to their symbolic and relational meaning. Therefore, the current results suggest that advocacy efforts by a university may by associated with favorable attitudes and behaviors among students when they are perceived as relevant, credible, and aligned with student values.

These findings should not be interpreted as mere statistical significance; the importance of these results lies in explaining the mechanisms by which CSA is associated with positive behavioral outcomes (i.e., CCB) in the higher education sector of Northern Cyprus. While advocacy initiatives can influence students’ behavior directly, mediating effects demonstrate that emotional attachment and online communicative expressions can help this influence become behaviorally meaningful and solid. However, our research design has limitations, which are further explained in the final section of the paper (see Section 8).

## 5. Conclusions

The results obtained in this research empirically support the influence of CSA activities of universities on the CCB of their students and reveal the non-negligible mediating effects of BA and EWOM on the CSA–CCB linkage. These results highlight the cognitive and emotional mechanisms shaped under CSA initiatives that lead to voluntary positive behavior among students. The contextual framework of the education sector of Northern Cyprus provides a unique setting for showcasing the current contributions. Notably, in the tourism and banking industries, CCB is conceptualized as transactional (Huang et al., 2023; Mohammad Shafiee & Tabaeeian, 2022). However, in the context of the education sector, CCB is more relational and value-centric, and is derived from socio-cultural communication and responses. For the case of a small island economy, the current results show a significant interplay among the CSA activities of a university and the psychological elements that can lead to extra-role behaviors (i.e., CCB). The confirmation of our research hypotheses implies that socially active universities can create a deeper sense of advocacy among their students, driving feedback among younger generations who are socially conscious.

Designing CSA initiatives that are genuinely important to students and are not symbolic or superficial can yield trust, emotional closeness, and a sense of shared values in student–university interactions. This can also open pathways for students to share positive experiences (EWOM), which can serve as an additional avenue for generating positive perceptions via supportive behavior.

## 6. Theoretical Implications

The current findings are interpreted using the premise of ELM, which provides a framework for understanding how CSA may differentially engage students depending on their involvement level and value alignment without claiming to directly measure route-specific processing, as noted earlier (Petty & Cacioppo, 1986). The mediating role of BA represents the affective processing route that can be generated through CSA activities, leading to an enhanced behavioral outcome, particularly regarding CCB. Similarly, the mediating role of EWOM reflects how social validation and external cues can shape the behavior of consumers (i.e., students in this case) with respect to CSA initiatives deployed by the university. This dual-pathway interpretation of ELM suggests its conceptual applicability in corporate social activities, though the current study operationalizes ELM as a framework rather than a model whose routes are directly measured. The literature on CSR often distinguishes between the rational and emotional processing of information (Petty & Cacioppo, 1986). The use of the education industry as a context extends the usage of ELM from marketing and advertising to a more socially oriented setting that shapes the dynamics of citizenship behavior. This is in line with the ELM being applied to corporate ethics and brand activism perspectives, suggesting its adequacy in such contexts (Parcha & Kingsley Westerman, 2020; J. Y. Li et al., 2022).

The current findings are consistent with an ELM-based interpretation in which CSA messages may motivate varying levels of cognitive engagement among students, though the study does not directly measure central or peripheral route processing, and these interpretations remain theoretical rather than empirically demonstrated. While emotional connectedness is explained through attachment (i.e., BA), the notion of EWOM represents a social phenomenon formed through the validation of other peers. This elaboration is made possible by the ELM, which provides the theoretical underpinning of the current contributions to the literature. The hybridity of cognitive–affective dynamics, specifically for the case of the current research (i.e., university students on a small island), can be a major determinant of individuals’ social consciousness (S. Park et al., 2024), extending the applicability of ELM. Notably, this also reveals that ELM can be adapted to studies examining younger, digitally savvy audiences, further expanding its relevance in the context of the CSR and CSA literature.

## 7. Practical Implications

The current research also has several practical implications that can be useful for practitioners in the field of academia, particularly for universities in Northern Cyprus. Managers and directors can see the potential benefits of CSA initiatives and embedding such activities into their core strategic development. This can further enhance the institution’s communication channels to better target younger audiences (e.g., prospective students). Through establishing value-driven communications (i.e., CSA), universities can develop and improve the sense of loyalty among their students, and encouraging among-peer advocacy fosters a cooperative involvement between the university and its students. The marketing strategy of universities can also emphasize the concept of BA, which directs campaigns towards an alignment with the social concerns of students. This can help generate EWOM in online platforms, which can further amplify the reach of university campaigns and keep individuals engaged at high and low levels. Since the education sector differs from industries where loyalty frameworks are set, the current findings can aid in the strategic positioning of universities as socially aware and responsible entities. Ultimately, this can improve the link between the university and its students, which transcends the mere transactional interactions occurring routinely.

## 8. Limitations

Despite the previously noted contributions, the current study was hindered by several factors:

1. As the research was conducted in a quantitative manner using the PLS-SEM technique, the sample size was limited, affecting the accuracy of the data and the extent to which the results can be generalized.
2. The method by which the data was collected was cross-sectional, limiting the determination of changes across time.
3. The context of small islands as emerging economies, particularly in the Middle East, remains underexplored, limiting the overall theme of the research.
4. While the premise of ELM provides sufficient theoretical support for the current study, the framework could be expanded to become more comprehensive.
5. Self-reported data can have biases (i.e., response bias), which can influence the interpretation of the results.
6. A potential construct overlap exists between the EWOM items and the CCB-Advocacy items, as both capture positive communicative behavior toward the university in the online domain. While HTMT values confirm empirical distinctiveness (see Table 3), the conceptual boundary between organic online sharing (EWOM) and deliberate advocacy behavior (CCB-Advocacy) remains a limitation of the current operationalization. Future studies should consider refining item wording to more sharply delineate these constructs or employ qualitative methods to verify respondents’ subjective distinction between the two behaviors.
7. The cross-sectional design of this study precludes causal interpretation of the findings. While the structural paths are statistically supported, the directionality of relationships reflects theoretical assumptions rather than empirical causality.
8. The current research does not include cultural or social factors in its equations, which limits the scope of the research.

## 9. Future Research Directions

Future studies can expand the sample size and assess the generalizability of these findings. The sampling techniques and context-specific nature of this research limit its generalizability, despite the theoretical extension obtained in the results. Institution type, sampling frames, and student populations can also be addressed in future studies to address this limitation.

Longitudinal studies can be conducted in the future to address changes in the behavior of students before and after a certain CSA initiated by their university, or in other industries. This calls for additional research and analyses in this regard to obtain comparative empirical evidence.

Future studies can employ other theories for examining the relationship between CSA and CCB, along with EWOM and BA (e.g., social exchange theory, theory of planned behavior, and stimulus–organism–response theory). This can be addressed in future studies examining similar contextual settings.

Future studies employing longitudinal or experimental designs would allow for stronger causal inference. Scholars can additionally address the contributions of cultural and social elements with respect to CSA and citizenship behaviors.

## Figures and Tables

**Table 1:** Respondent characteristics.

	Frequency	Percentage
Age		
18–21	86	39.3%
22–26	85	38.8%
27–30	32	14.6%
Over 30	16	7.3%
Gender		
Male	118	53.9
Female	101	46.1
Education		
Bachelor	152	69.4%
Masters	58	26.5%
PhD	9	4.1%

**Table 2:** Measurement model.

Construct	Indicators	Outer Loadings	Cronbach α	Rho_A	CR	AVE
**Corporate Social Advocacy**	CSA1	0.751	0.810	0.812	0.869	0.624
	CSA2	0.754				
	CSA3	0.762				
	CSA4	0.802				
**Brand Attachment**	BA1	0.821	0.844	0.847	0.906	0.762
	BA2	0.805				
	BA3	0.852				
**EWOM**	EW1	0.862	0.876	0.879	0.921	0.657
	EW2	0.763				
	EW3	0.803				
**CCB—Helping**	HP1	0.841	0.821	0.824	0.883	0.716
	HP2	0.809				
	HP3	0.857				
**CCB—Advocacy**	AD1	0.811	0.813	0.816	0.888	0.726
	AD2	0.806				
	AD3	0.732				
**CCB—Feedback**	FB1	0.859	0.794	0.796	0.879	0.707
	FB2	0.863				
	FB3	0.721				

**Table 3:** Heterotrait–Monotrait ratio (HTMT).

	CSA	BA	EW	HP	AD
**CSA**	-				
**BA**	0.721	-			
**EW**	0.465	0.512	-		
**HP**	0.711	0.614	0.717	-	
**AD**	0.602	0.679	0.644	0.687	-
**FB**	0.633	0.644	0.651	0.677	0.725

**Table 4:** Higher-order construct assessment: CCB (Type II reflective–formative).

Construct	Dimensions	Convergent Validity	Weights	VIF	t-Statistics
**CCB**	Helping	0.717	0.362	1.919	4.051
	Advocacy		0.366	1.705	4.014
	Feedback		0.323	1.776	4.089

**Table 5:** Hypothesis testing.

Effects	Relations	β	t-Statistics	ƒ^2^	Decision
**Direct**					
**H1**	CSA → CCB	0.323	4.678 **	0.101	Supported
**Mediation**					
**H2**	CSA → BA → CCB	0.317	2.844 *	0.104	Supported
**H3**	CSA → EWOM → CCB	0.342	2.895 *	0.111	Supported
**Control Variables**					
	Gender → CCB	0.124	2.514 *		
	Age → CCB	0.113	2.132 *		

* *p* < 0.05 and ** *p* < 0.01. R^2^BA = 0.35/Q^2^BA = 0.16/RMSE = 0.667/MAE = 0.380 R^2^EWOM = 0.48/Q^2^EWOM = 0.26/RMSE = 0.671/MAE = 0.387 R^2^CCB = 0.51/Q^2^CCB = 0.37/RMSE = 0.675/MAE = 0. 391 SRMR = 0.061.

## Data Availability

Data availability is not applicable for this study as no new data were generated or analyzed.

## Abbreviation

The following abbreviations are used in this manuscript:

CSA	Corporate Social Advocacy
ELM	Elaboration Likelihood Model
CCB	Customer Citizenship Behavior
BA	Brand Attachment
EWOM	Electronic Word-of-Mouth
SIDS	Small Island Developing States
CSR	Corporate Social Responsibility
PLS-SEM	Partial Least Squares Structural Equation Modeling
AVE	Average Variance Extracted
VIF	Variance Inflation Factor

## Appendix A. Survey Instrument

All items measured on a 5-point Likert scale (1 = Strongly Disagree, 5 = Strongly Agree).

Corporate Social Advocacy (CSA) (Hambrick & Wowak, 2021; M. D. Dodd & Supa, 2014):

- CSA1: My university actively takes a stance on important social and political issues.
- CSA2: My university advocates for social causes that go beyond its core academic mission.
- CSA3: My university’s position on social issues aligns with my personal values.
- CSA4: My university engages in advocacy that addresses issues relevant to society.
- Brand Attachment (BA) (C. W. Park et al., 2010)
- BA1: I feel a strong sense of connection to my university.
- BA2: My university is part of who I am.
- BA3: I feel personally attached to my university.

Electronic Word-of-Mouth (EWOM) (Hennig-Thurau et al., 2004; Cheung & Thadani, 2012):

- EW1: I share positive information about my university on social media or online platforms.
- EW2: I recommend my university to others through online channels.
- EW3: I post positive content about my university’s activities and initiatives online.

Customer Citizenship Behavior—Helping (Yi & Gong, 2013):

- HP1: I assist other students who need help navigating university services.
- HP2: I help new students when they face difficulties.
- HP3: I willingly share useful information with fellow students.

Customer Citizenship Behavior—Advocacy (Groth, 2005; Yi & Gong, 2013):

- AD1: I actively promote my university to people I know.
- AD2: I say positive things about my university to others.
- AD3: I encourage others to consider enrolling at my university.

Customer Citizenship Behavior—Feedback (Yi & Gong, 2013):

- FB1: I provide suggestions to my university to help improve its services.
- FB2: I inform the university when something could be done better.
- FB3: I give constructive feedback to help my university grow.
